# Global Burden of Anxiety and Depression among Cystic Fibrosis Patient: Systematic Review and Meta-Analysis

**DOI:** 10.1155/2021/6708865

**Published:** 2021-07-07

**Authors:** Mistire Teshome Guta, Tiwabwork Tekalign, Nefsu Awoke, Robera Olana Fite, Getahun Dendir, Tsegaye Lolaso Lenjebo

**Affiliations:** ^1^School of Nursing, College of Health Science and Medicine, Wolaita Sodo University, Wolaita Sodo, Ethiopia; ^2^HaSET Program/Ethiopian Public Health Institute, Adis Abeba, Ethiopia; ^3^School of Anesthesia, College of Health Science and Medicine, Wolaita Sodo University, Wolaita Sodo, Ethiopia; ^4^School of Public Health, College of Health Science and Medicine, Wolaita Sodo University, Ethiopia

## Abstract

**Aims:**

This systemic review and meta-analysis were aimed at determining the level of anxiety and depression among cystic fibrosis patients in the world.

**Methods:**

We conducted a systematic search of published studies from PubMed, EMBASE, MEDLINE, Cochrane, Scopus, Web of Science, CINAHL, and manually on Google Scholar. This meta-analysis follows the Preferred Reporting Items for Systematic Reviews and Meta-Analyses (PRISMA) guidelines. The quality of studies was assessed by the modified Newcastle-Ottawa Scale (NOS). Meta-analysis was carried out using a random-effects method using the STATA™ Version 14 software. Trim and fill analysis was done to correct the presence of significant publication bias.

**Result:**

From 419,820 obtained studies, 26 studies from 2 different parts of the world including 9766. The overall global pooled prevalence of anxiety and depression after correction for publication bias by trim and fill analysis was found to be 24.91(95% CI: 20.8-28.9) for anxiety. The subgroup analyses revealed with the lowest prevalence, 23.59%, (95% CI: 8.08, 39.09)) in North America and the highest, 26.77%, (95% CI: 22.5, 31.04) seen in Europe for anxiety and with the highest prevalence, 18.67%, (95% CI: 9.82, 27.5) in North America and the lowest, 13.27%, (95% CI: -10.05, 16.5) seen in Europe for depression.

**Conclusion:**

The global prevalence of anxiety and depression among cystic fibrosis patients is common. Therefore, close monitoring of the patient, regularly screening for anxiety and depression, and appropriate prevention techniques is recommended.

## 1. Background

Depression is a mental disorder characterized by feelings of depressed mood, loss of interest or pleasure in activities, and loss of energy that lasts for 2 weeks or more [1]. Depression disorder presents with depressed mood, loss of interest or pleasure, decreased energy, feelings of guilt or low self-worth, disturbed sleep or appetite, poor concentration, the problem of thinking and making decisions, and, in severe stages, recurring thoughts of death or suicide [2], whereas anxiety is a vague, subjective, nonspecific feeling of uneasiness, apprehension, tension, (excessive nervousness) fears, and a sense of impending doom, irrational avoidance of objects or situation, and anxiety attack [3]. Anxiety and depression are the most frequently occurring mental disorders in the general population [4]. Moreover, depression often comes with symptoms of anxiety. These problems can become chronic or recurrent and lead to substantial impairments in an individual's ability to take care of his or her everyday responsibilities [5].

According to the WHO report in 2017, 300 million people around the world have depression and also, the burden of depression and other mental health conditions is on the rise globally [6]. Similarly in another study, globally more than 264 million people affected with depression [7]. The high prevalence suggests that immediate preventive measures should be implemented, such as the setting up of psychopedagogic support services for those in need [8]. Moreover, depression is a significant determinant of quality of life and survival, accounting for approximately 50% of psychiatric consultations and 12% of all hospital admissions [9]. Cystic fibrosis (CF) is a common genetic, life-shortening chronic illness, leading to frequent infections and progressive failure of most organ systems (e.g., lungs and pancreas) [10]. The disease is highly burdensome with progressive multisystem nvolvement, mostly problematic due to persistent lung infections, frequent hospitalizations, and time-consuming treatment regimens taking 2 to 4 hours per day on average [11, 12]. It is a severe and progressive disease characterized by decreased physical activities and excessive dyspnea on exertion [13]. Chronically ill patients experience emotional difficulties that can sometimes even manifest themselves in anxiety and depression [14]. The study showed that individuals with chronic medical conditions have a 41% increased risk of having a psychiatric disorder [15]. Respiratory diseases have an increased risk for comorbid anxiety and depression [16]. Depression, anxiety, and cognitive impairment are common among patients with chronic obstructive pulmonary disease (COPD) and may both affect the delivery of pulmonary rehabilitation and be modified by pulmonary rehabilitation [17]. Studies show that compared with nondepressed patients, the odds are 3 times greater than depressed patients that will be noncompliant with medical treatment recommendations [18]. Symptoms of depression and anxiety have negative consequences for disease management, health-related quality of life, and health outcomes [19]. Studies measuring psychological distress in individuals with CF have found high rates of both depression and anxiety, and the prevalence of depression and anxiety ranges from 13–33% and 30% to 33% among adults, respectively [20, 21]. Depressive symptoms are prevalent among adults with CF and are associated with poorer health-related quality of life even after controlling for the lung function [14]. The study indicated that people with CF and parents who take care of children with CF are more likely to experience depression than people in the general population [22]. Since the presence of depression and anxiety has a negative influence on the quality of life, healthcare cost, and self-care, this meta-analysis contributes its own to give attention to CF patients especially to focus on prevention and screen for depression and anxiety and treat accordingly.

## 2. Methods and Materials

### 2.1. Study Design and Search Strategy

This systematic review and meta-analysis were conducted under the Preferred Reporting Items guidelines for Systematic Reviews and Meta-analyses (PRISMA) statement [23, 24]. We made the searching of articles published in the English language regardless of the year of publication that was taken from PubMed, EMBASE, MEDLINE, Cochrane, Scopus, the web of Science CINAHL, and manually on Google Scholar. The search was performed using key terms such as CF, cystic fibrosis, anxiety, depression, patient, and worldwide.

### 2.2. Study Selection and Eligibility Criteria

- All adults who were diagnosed with CF were included
- Both published and unpublished studies conducted worldwide regardless of study design were included

### 2.3. Study Extraction and Quality Appraisal

The data were extracted by three independent authors (MT, TT, and NA) using a data extraction format prepared in a Microsoft Excel 2010 spreadsheet. The extracted data were the first author's name, publication year, country, region, design, sample size, sampling method, and prevalence of anxiety and depression among CF patient. The quality of each study was assessed using the modified Newcastle-Ottawa Scale (NOS) for cross-sectional studies [25]. Studies were included with a score of 5 and more on the NOS [26]. Each study's quality was evaluated independently by five authors (TT, MT, RO, GD, and TL), and any disagreements were resolved by discussion and consensus.

### 2.4. Publication Bias and Heterogeneity

To assess the existence of publication bias, funnel plots were used, and Egger's test was computed. A *p* value<0.05 was used to declare the statistical significance of publication bias. *I*^2^ test statistics were used to check the heterogeneity of studies. *I*^2^ test statistics of <50, 50–75%, and>75% were declared as low, moderate, and high heterogeneity, respectively [27].

### 2.5. Outcome Measure

The outcome of this review was the global prevalence of anxiety and depression among CF patient.

### 2.6. Data Synthesis and Analysis

STATA™ Version 14software was used to conduct the analysis. The heterogeneity test was conducted by using *I*−squared (*I*^2^) statistics. The pooled prevalence of anxiety and depression among CF patient was carried out using a random-effects (DerSimonian and Laird) method. To minimize the potential random variations between studies, heterogeneity sources were analyzed using subgroup analysis and metaregression.

## 3. Results

### 3.1. Study Selection

**Figure 1:**
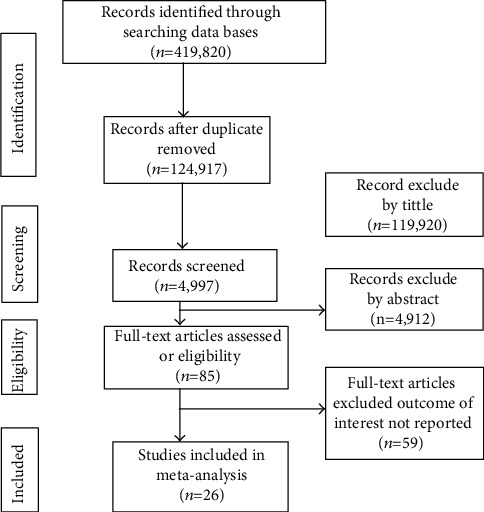
PRISMA flow diagram of study selection.

Initially, a total of 419,820 studies were retrieved from the databases and manual searching. From this, 124,917 duplicates were found and removed. Their title screened the remaining 119,920 articles, and abstract 4,912 irrelevant studies were removed. Eighty-five full-text articles were assessed for eligibility, and 59 of them were excluded due to not reporting the outcome of interest and low methodological quality. Finally, a total of 26 studies fulfilled the inclusion criteria and enrolled in the study (Figure 1).

### 3.2. Study Characteristics

Twenty-four studies for anxiety included 9567 participants, and 26 studies for depression included 9766 [22, 28–42]. All of the included studies were cross-sectional studies, and the sample size ranged from 22 [28] to 2042 [22]. Most studies were conducted in Europe. Among the included studies, the prevalence of anxiety and depression among cystic fibrosis patient ranges from 5 [40] to 46 [38] and from 6 [39] to 45 [38], respectively (Table 1).

### 3.3. The Prevalence of Anxiety and Depression in Patients with Cystic Fibrosis

**Figure 2:**
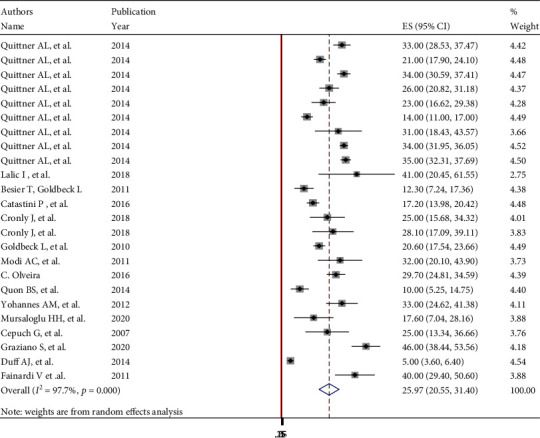
Forest plot showing global pooled prevalence of anxiety in patients with cystic fibrosis.

**Figure 3:**
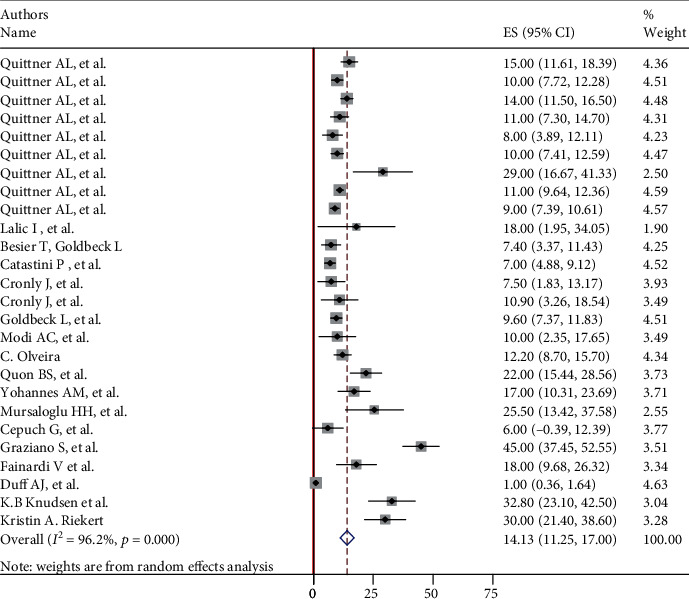
Forest plot showing global pooled prevalence of depression in patients with cystic fibrosis.

The overall pooled global prevalence of anxiety and depression in patients with cystic fibrosis was 26.22% (95% CI: 22.1, 30.2) and 14.13% (95% CI: 11.25, 17.0) with a heterogeneity index (*I*^2^) of 93.5% and 96.2%, respectively, *p* < 0.001 (Figures 2 and 3). And since the Eggers test was found significant, the final pooled prevalence was corrected for Duval and Tweedie's trim and fill analysis and was found to be 24.91 (95% CI: 20.8-28.9) for anxiety but for depression, it is similar.

### 3.4. Subgroup Analysis of Anxiety

**Figure 4:**
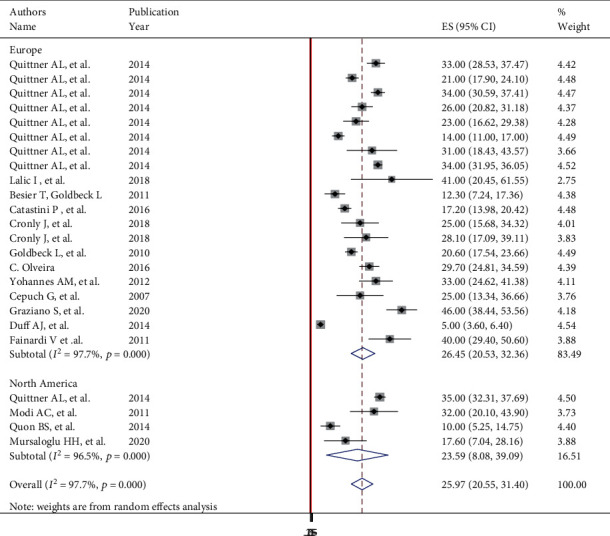
Subgroup analysis of global prevalence of anxiety in patients with cystic fibrosis.

Subgroup analyses revealed a marked variation across the continents, with the lowest prevalence 23.59% (95% CI: 8.08, 39.09), *I*^2^ = 96.5%) in North America and the highest 26.77% ((95% CI: 22.5, 31.04), *I*^2^ = 92.8%) seen in Europe (Figure 4).

### 3.5. Subgroup Analysis of Depression

**Figure 5:**
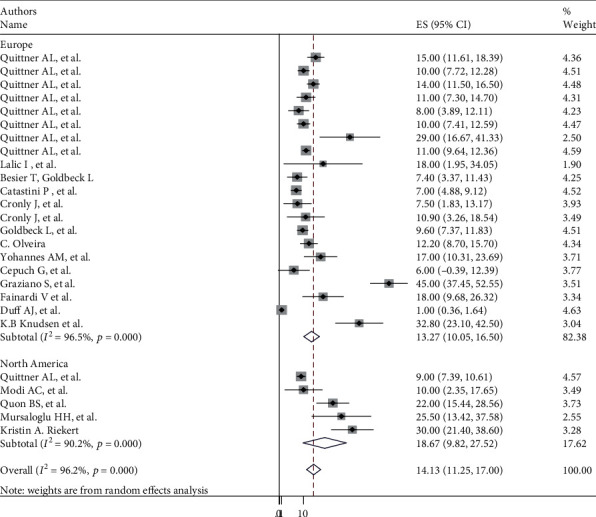
Subgroup analysis of global prevalence of depression in patients with cystic fibrosis.

Subgroup analyses revealed a marked variation across the continents, with the highest prevalence 18.67% (95% CI: 9.82, 27.5), *I*^2^ = 90.2%) in North America and the lowest 13.27% (95% CI: -10.05, 16.5), *I*^2^ = 96.5%) seen in Europe (Figure 5).

### 3.6. Metaregression

Metaregression was conducted using the year of publication and sample size as a covariate to identify the source of heterogeneity. It was indicated that there is no effect of publication year and sample size on heterogeneity between studies (Tables 2 and 3).

### 3.7. Publication Bias

**Figure 6:**
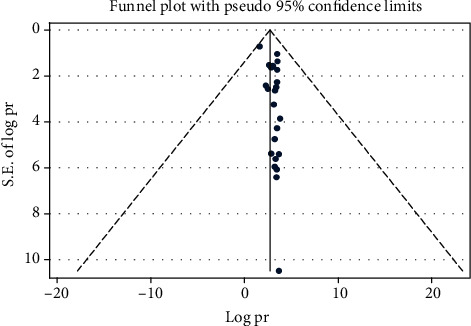
Funnel plot to test the publication bias in 24 studies of anxiety with 95% confidence limits.

**Figure 7:**
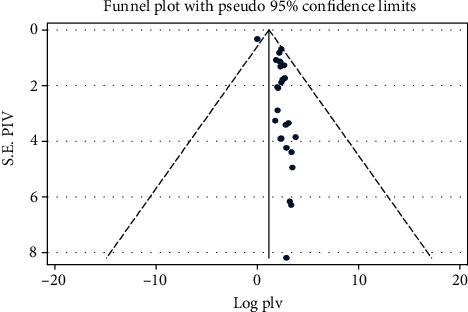
Funnel plot to test the publication bias of depression in 26 studies with 95% confidence limits.

The presence of publication bias was evaluated graphically by funnel plots for both anxiety and depression. Both plots indicate paper asymmetrical. We have tested statically for the presence of small study effect and effect of unpublished study for both anxiety and depression. The egger test for anxiety relived there is presence of small study effect with *p* < 0.001, and the Begg test indicates there is no statistical evidence for presence of unpublished studies with *p* value >0.05. The egger test for depression showed no small study effect with *p* < 0.001, and we have tested for the presence of unpublished study effect for depression by the begg test and since *p* value is >0.05, there is no statistical evidence for presence of unpublished studies (Figures 6 and 7) .

## 4. Discussion

Primary studies suggest that CF does have a negative impact on physical functioning aspects of quality of life. Depression and anxiety are the main factors in decreasing individual's quality of life. The prevalence of anxiety and depression among adults with cystic fibrosis patients found to be 22.2% and 42.4%, respectively [14]. In another finding, the cystic fibrosis patients have significantly worse quality of life reflecting their significantly impaired scores for physical functioning [43]. Similarly, young adult CF patients have lower scores on several measures of physical functioning and for general health perception, but to have similar scores for most psychosocial measures [44, 45]. Since Cystic fibrosis is a genetic disorder that damages many of the body's organs, on the top of medical treatment psychological treatment also helps to reduce anxiety and depression, improve adjustment, quality of life, and even medical outcomes, as well as knowledge, skills, and decisions regarding care.

This systematic review and meta-analysis estimated the pooled global prevalence of anxiety and depression among cystic fibrosis patients. Twenty-two studies were included in the analysis, which selected based on an inclusion criterion. To the best of our knowledge, this systematic review and meta-analysis provide a comprehensive estimation of the global pooled prevalence of anxiety and depression among cystic fibrosis patients. Depression and anxiety are more commonly observed in chronic diseases compared to the general population [46].

In this systematic review and meta-analysis, the overall pooled prevalence of anxiety and depression among cystic fibrosis patients was 26.22% (95% CI: 22.1, 30.2) and 12.66% (95% CI: 10.6, 14.6), respectively. This suggests that anxiety and depressive symptoms are common in CF patients, and it needs intensive and multifactorial approach that is required to combat the CF-related complications. Since CF is a life-threatening and incurable chronic medical disease, untreated anxiety and depression have a substantial impact on CF patients' quality of life, physical function, and healthcare utilization. Routine screening for symptoms of anxiety and depression is a worthy endeavor, and those identified with elevated clinical symptoms should be referred to receive appropriate treatment. This systematic review and meta-analysis have an implication for the health professional, patients, and patient's family.

In this systematic review and meta-analysis, we had the highest prevalence of anxiety among CF patients in Italia, 46% (95% CI: 38.44, 53.56), and lowest in North America, 10% (95% CI: 5.25, 14.75), whereas 45% (95% CI: 37.45, 52.55) in Italia and 6% (95% CI: -0.39, 12.39) in Poland, respectively, had prevalence of depression among CF patients. In subgroup analysis in Europe, we had 46% (95% CI: 38.44, 53.56) which has the highest prevalence of anxiety among CF patients in Italia and lowest in Germany, 12.3% (95% CI: 7.24, 17.36), whereas in Northern America, 35% (95% CI: 32.31, 37.69) and 10% (95% CI: 5.25, 14.75), respectively. For the highest prevalence of depression among CF patients in Europe, it was 45% (95% CI: 37.45, 52.55) in Italia and lowest in Poland, 6% (95% CI: -0.39, 12.39), whereas in North America, it is 25.5% (95% CI: 13.42, 37.58) and 9% (95% CI: 7.39, 10.61), respectively.

The strength of the systematic review and meta-analysis is the use of an extensive search strategy to incorporate the studies. On the other hand, studies are reported from a limited number of the country in the world and that may create underrepresentation. This systematic review and meta-analysis presented up-to-date evidence on the global prevalence of anxiety and depression among cystic fibrosis patients.

This systematic review and meta-analysis might have faced the following limitations. First, the lack of primary studies other than Europe and northern America and may affect the generalizability of the finding. Secondly, due to there is significant heterogeneity and publication bias, the result needs to be interpreted cautiously. Thirdly, using different scales of the primary studies included in this systematic review and meta-analysis for screening depression and anxiety among CF patients might affect the pooled prevalence. Our final limitation is having difficulties in comparing our finding due to a lack of summarized and regional-wide systematic reviews and meta-analysis on the prevalence of anxiety and depression among CF patient.

## 5. Conclusion

The global prevalence of anxiety and depression among CF patient is common. Therefore, close monitoring of the patient, regularly screening for anxiety and depression and appropriate prevention techniques is recommended.

## Figures and Tables

**Table 1:** Characteristics of the included studies of anxiety in the systematic review and meta-analysis.

Authors' name	Publication year	Study area	Region	Study design	Sample	Prevalence of anxiety % (95% CI)	Prevalence of depression % (95% CI)
Quittner et al.	2014	Belgium	Europe	Cross-sectional	426	33 (28.53-37.46)	15 (11.61, 18.39)
Quittner et al.	2014	Germany	Europe	Cross-sectional	663	21 (17.90, 24.10)	10 (7.72, 12.28)
Quittner et al.	2014	Italy	Europe	Cross-sectional	741	34 (30.59, 37.18)	14 (11.50, 16.50)
Quittner et al.	2014	Spain	Europe	Cross-sectional	275	26 (20.82, 31.18)	11(7.30,14.70)
Quittner et al.	2014	Sweden	Europe	Cross-sectional	167	23 (16.62, 29.68)	8 (3.89, 12.11)
Quittner et al.	2014	Netherlands	Europe	Cross-sectional	515	14 (11.00, 17.00)	10 (7.41, 12.59)
Quittner et al.	2014	Turkey	Europe	Cross-sectional	52	31 (18.43, 43.57)	29 (16.67, 41.33)
Quittner et al.	2014	United Kingdom	Europe	Cross-sectional	2042	34 (31.95, 36.05)	11 (9.64, 12.36)
Quittner et al.	2014	United States	North America	Cross-sectional	1207	35 (32.31, 37.69)	9 (7.39, 10.61)
Lalic et al.	2018	Croatia	Europe	Cross-sectional	22	41(20.45,61.45)	18(1.95,34.05)
Besier and Goldbeck	2011	German	Europe	Cross-sectional	162	12.3 (7.24, 17.36)	7.4 (3.37, 11.43)
Catastini et al.	2016	Italia	Europe	Cross-sectional	528	17.2 (13.98, 20.42)	7 (4.88, 9.12)
Cronly et al.	2018	Ireland	Europe	Cross-sectional	83	25 (15.68, 34.32)	7.5 (1.83, 13.17)
Cronly et al.	2018	Ireland	Europe	Cross-sectional	64	28.1 (17.09, 39.11)	10.9(3.26, 18.54)
Goldbeck et al.	2010	German	Europe	Cross-sectional	670	20.6 (17.54, 23.66)	9.6 (7.37, 11.83)
Modi et al.	2011	USA	North America	Cross-sectional	59	32 (20.10, 43.90)	10 (2.35, 17.65)
Olveira	2016	Spain	Europe	Cross-sectional	336	29.7 (24.81, 34.59)	12.2(8.70,15.70)
Quon et al.	2014	USA	North America	Cross-sectional	153	10 (5.25, 14.75)	22 (15.44, 28.56)
Yohannes et al.	2012	United Kingdom	Europe	Cross-sectional	121	33 (24.62, 41.38)	17(10.31,23.69)
Mursaloglu et al.	2020	USA	North America	Cross-sectional	50	17.6 (7.04, 28.16)	25.5 (13.42, 37.58)
Cepuch et al.	2007	Poland	Europe	Cross-sectional	53	25 (13.34, 36.66)	6 (-0.39, 12.39)
Graziano et al.	2020	Italia	Europe	Cross-sectional	167	46 (38.44, 53.56)	45 (37.45, 52.55)
Fainardi et al.	2011	Italia	Europe	Cross-sectional	82	40 (89.23-186.54)	18 (9.68-26.31)
Duff et al.	2014	United Kingdom	Europe	Cross-sectional	929	5 (932.15-1819.65)	10 (0.36-1.63)
Knudsen et al.	2016	Denmark	Europe	Cross-sectional	90	—	32.8 (23.1-42.4)
Riekert	2007	USA	North America	Cross-sectional	109	—	30 (21.39-38.6)

**Table 2:** Metaregression analysis of factors affecting between-study heterogeneity of depression.

Heterogeneity source	Coefficients	Std. err.	*p* value
Publication year	0.3057765	0.6975618	0.665
Sample size	0.0058508	0.0041811	0.175

**Table 3:** Metaregression analysis of factors affecting between-study heterogeneity of anxiety.

Heterogeneity source	Coefficients	Std. err.	*p* value
Publication year	0.2517433	1.581655	0.875
Sample size	0.011806	0.0084867	0.179

## Data Availability

Datasets are available through the corresponding author upon rational appeal.

## Abbreviations

CI: Confidence interval
NOS: Newcastle Ottawa Scale
OR: Odds ratio
PRISMA: Preferred Reporting Items for Systematic Reviews and Meta-Analyses.
